# Improvement of angiographic and clinical outcomes of percutaneous coronary intervention for chronic total occlusion after implementation of a dedicated team: a single-centre experience

**DOI:** 10.1007/s12471-022-01732-5

**Published:** 2022-11-29

**Authors:** L. Polimac, M. M. C. J. van Leunen, G. J. van Steenbergen, J. M. Zelis, R. Eerdekens, M. van ’t Veer, D. N. Schulz, I. F. Wijnbergen, P. J. Vlaar, K. Teeuwen

**Affiliations:** Heart Centre, Catharina Hospital, Eindhoven, The Netherlands

**Keywords:** Chronic total occlusion, Percutaneous coronary intervention, Hybrid algorithm

## Abstract

**Introduction:**

In a Dutch heart centre, a dedicated chronic total occlusion (CTO) team was implemented in June 2017. The aim of this study was to the evaluate treatment success and clinical outcomes before and after this implementation.

**Methods:**

A total of 662 patients who underwent percutaneous coronary intervention (PCI) for a CTO between January 2013 and June 2020 were included and divided into pre– and post–CTO team groups. The primary endpoint was the angiographic success rate of CTO-PCI. Secondary endpoints included angiographic success stratified by complexity using the J‑CTO score and the following clinical outcomes: in-hospital complications and myocardial infarction, target vessel revascularisation, all-cause mortality, quality of life (QoL) and major adverse cardiac events (MACE) at 30-day and 1‑year follow-up.

**Results:**

Compared with the pre–CTO team group, the success rate in the post–CTO team group was higher after the first attempt (81.4% vs 62.7%; *p* < 0.001) and final attempt (86.7% vs 73.8%; *p* = 0.001). This was mainly driven by higher success rates for difficult and very difficult CTO lesions according to the J‑CTO score. The MACE rate at 1 year was lower in the post–CTO team group than in the pre–CTO team group (6.4% vs 16.0%; *p* < 0.01), while it was comparable at 30-day follow-up (0.1% vs 1.7%; *p* = 0.74). Angina symptoms were significantly reduced at 30-day and 1‑year follow-up, and QoL scores were higher after 1 year.

**Conclusion:**

This study demonstrated higher success rates of CTO-PCI and improved clinical outcomes and QoL at 1‑year follow-up after implementation of a dedicated CTO team using the hybrid algorithm.

**Supplementary Information:**

The online version of this article (10.1007/s12471-022-01732-5) contains supplementary material, which is available to authorized users.

## What’s new?

- Centralising percutaneous coronary intervention (PCI) for chronic total occlusion (CTO) by assigning this procedure to dedicated trained operators who used hybrid approach led to higher angiographic success rates and improved clinical outcomes.
- The higher angiographic success rate of CTO-PCI performed by the CTO team was most prominent for difficult and very difficult CTO lesions.
- Despite increasing CTO complexity, the rate of periprocedural complications seen by the CTO team remained acceptable, while the incidence of major adverse cardiac events was reduced at 1‑year follow-up.
- Angina symptoms were significantly reduced at 30-day and 1‑year follow-up, and QoL scores were higher after 1 year.

## Introduction

Chronic total occlusions (CTOs) are observed in almost 20% of all patients diagnosed with coronary artery disease [1]. Historically, CTOs were the ‘Achilles heel’ of percutaneous coronary intervention (PCI), with lower rates of procedural success, higher complication rates and higher risk of restenosis compared with nonocclusive lesions [2]. In the past century, antegrade wire escalation was the sole technique for CTO-PCI, with a success rate of only 70% in experienced hands [3].

Technical success rates of CTO-PCI have been rising due to the development of novel revascularisation techniques and adoption of a hybrid algorithm [4–7]. As a result, three additional recanalisation techniques have evolved: antegrade dissection and re-entry, retrograde wire escalation, and retrograde dissection and re-entry. Furthermore, the hybrid algorithm allows interventional cardiologists to combine several techniques within one procedure to achieve higher success rates [4, 7, 8]. Nowadays, by using the hybrid algorithm high procedural success rates (> 90%) and an acceptable incidence of complications (2.3%–4.3%) can be achieved by dedicated and experienced operators [9–11].

In the Catharina Hospital in Eindhoven, the Netherlands, a dedicated CTO team of three interventional cardiologists, who have been trained in all CTO revascularisation techniques and the hybrid algorithm, was established. The aim of this study was to investigate the effect of implementation of a dedicated CTO team in a single centre on procedural results and clinical outcomes.

## Methods

### Implementation of CTO team

In June 2017, the CTO team was implemented, made up of three interventional cardiologists who were dedicated to perform CTO-PCI. Before this date, all staff members performed this procedure. The dedicated CTO interventional cardiologists received full training and carried out multiple hands-on CTO-PCI procedures in patients under direct supervision of highly experienced CTO expert operators. Furthermore, a complete CTO state-of-the-art equipment set was collected and stored in a mobile storage cabinet. This was present during every procedure in the operating room, thereby enabling a fast exchange of dedicated materials, which is mandatory for a successful hybrid approach.

All catheterisation laboratory nurses were trained in CTO recanalisation techniques, use of the materials and complication management. Prior to the procedure, each CTO case was discussed by at least two interventional cardiologists of the CTO team to determine the Multicentre CTO Registry of Japan (J-CTO) score and the appropriate approach according to the hybrid algorithm. All procedures for cases with higher complexity (J-CTO score > 3) or for previously failed cases were scheduled with two interventional cardiologists (‘double scrub’).

Prior to June 2017, patients were indicated for CTO-PCI if they presented with angina and had proven ischaemia. In June 2017, we changed these criteria to the presence of angina and viability (instead of ischaemia) as shown by normal wall motion on any imaging modality [12]. In case of hypokinetic or akinetic wall motion in the myocardial area of a CTO, additional viability testing with either single-photon emission computed tomography, magnetic resonance imaging or positron emission tomography was required. Patients’ quality of life (QoL) was routinely assessed using the Short Form Health Survey (SF-36) from 2018 onward.

### Study population

All patients who underwent CTO-PCI between January 2013 and June 2020 were included and divided into two groups: the pre–CTO team group and the post–CTO team group. Patients could be included in both Pre -and Post-CTO team group, if the previous target vessel CTO-PCI failed and was repeated at least 1 year after the first attempt. All patients in whom with CTO-PCI was performed by a non–CTO team member after June 2017, were excluded. Ethical approval was obtained by the local ethics committee.

### Endpoints, data collection and definitions

The primary endpoint was defined as the angiographic success rate of CTO-PCI, either at the first attempt or at subsequent attempts. The first secondary endpoint was the angiographic success rate of CTO-PCI, stratified by complexity according to the J‑CTO score. Additional secondary endpoints comprised the following clinical outcomes: in-hospital and procedural complications (consisting of myocardial infarction (MI), all-cause mortality, urgent pacemaker implantation, urgent cardiac surgery, stroke and in-stent thrombosis), and MI, target vessel revascularisation (TVR), all-cause mortality, QoL and major adverse cardiac events (MACE) at 30-day and 1‑year follow-up.

Baseline and procedural data were collected from electronic patient files. Angiographic endpoints were assessed and adjudicated by two independent experienced reviewers (LP, ML) and a third reviewer (KT) in case of disagreement. All angiographic and clinical endpoint definitions are listed in Table S1 (see Electronic Supplementary Material).

### Statistical analysis

Baseline characteristics of the two groups were compared using the unpaired *t*-test or Mann-Whitney U test for numerical variables and chi-square or Fisher’s exact test for categorical variables. Data are expressed as mean ± standard deviation for numerical variables and as number (percentage) for categorical variables. Analysis of angiographic success was performed on a lesion/procedural level.

Event-free survival curves for adverse cardiac events were estimated using the Kaplan-Meier method and compared with the log-rank test. A multivariate Cox proportional hazard analysis was performed. The Canadian Cardiovascular Society grading of angina pectoris (CCS) classification progress was compared using the Wilcoxon signed-rank test. For both the group with angiographic successful CTO-PCI and the group with angiographic unsuccessful CTO-PCI, baseline scores on 8 QoL domains were compared with the follow-up scores on these domains. For inclusion, the SF-36 questionnaire had to be completed both at baseline and at 1‑year follow-up and at least 50% of the items of each domain had to be answered.

A two-tailed probability value of < 0.05 was considered statistically significant. All statistical analyses were performed with IBM SPSS Statistics version 24.0 (IBM Corp., Armonk, NY, USA).

## Results

### Baseline characteristics

**Fig. 1:**
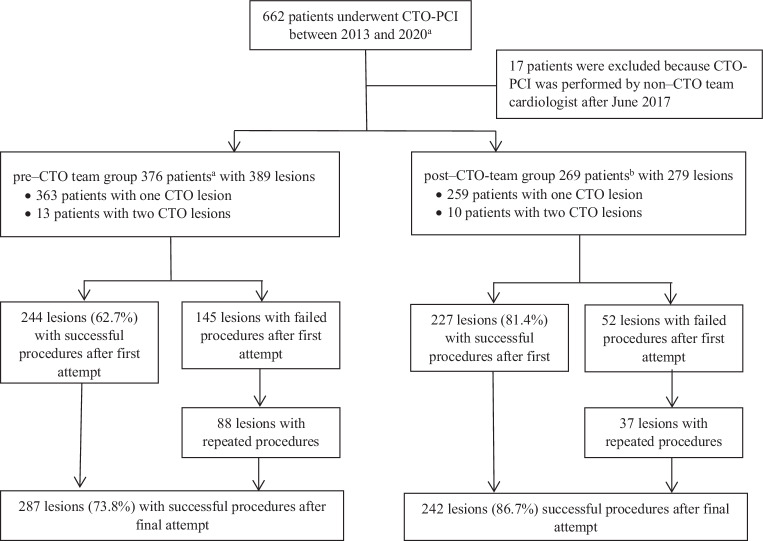
Flowchart of patient inclusion. ^a^ Seven patients were included in both groups. ^b^ One patient underwent two percutaneous coronary interventions (*PCIs*) for chronic total occlusion (*CTO*) procedures in the post–CTO team group for the same lesion

During the study period, a total of 662 patients underwent CTO-PCI, of whom 376 (58.3%) with 389 lesions were included in the pre–CTO team group and 269 (41.7%) patients with 279 lesions in the post–CTO team group; 17 patients were excluded because a non–CTO team interventional cardiologist had performed the procedure after June 2017. Seven patients were included in both groups, and one patient was included twice in the post-CTO team group (Fig. 1). Baseline characteristics were comparable, except for hypertension and age (Tab. 1; and Tables S2 and S3 in Electronic Supplementary Material). The mean J‑CTO score was comparable between both groups (2.00 vs 1.97; *p* = 0.64).

**Table 1:** Baseline characteristics, angiographic characteristics and procedural characteristics^a^

Variable	Pre–CTO team (*n* = 376)	Post–CTO team (*n* = 269)	*P*-value
*Baseline characteristics*			
Age, years	64.2 ± 10.8	66.9 ± 10.3	< 0.01
Male	291 (77.6)	215 (79.3)	0.63
Diabetes mellitus	86 (22.9)	60 (22.1)	0.85
Hypertension	244 (65.1)	220 (81.5)	< 0.01
Hypercholesterolaemia	336 (89.6)	232 (86.2)	0.21
Family history of CVD	129 (55.6)	101 (60.1)	0.41
Chronic kidney impairment	41 (11.0)	43 (15.9)	0.08
Previous myocardial infarction	179 (47.7)	111 (41.0)	0.09
Previous PCI	162 (43.2)	117 (43.2)	1.00
Previous CABG	87 (23.2)	62 (22.9)	1.00
Previous peripheral artery disease	28 (7.5)	26 (9.6)	0.39
Previous stroke	27 (7.2)	21 (7.7)	0.88
Atrial fibrillation	40 (10.7)	35 (12.9)	0.39
Left ventricular function			0.08
> 50%	272 (73.5)	209 (79.8)	
35%–50%	65 (17.6)	41 (15.6)	
< 35%	33 (8.9)	12 (4.6)	
	389 procedures	279 procedures	
*Angiographic characteristics*			
Occluded target vessel			< 0.01
RCA	181 (46.5)	142 (50.9)	
LAD	116 (29.8)	90 (32.3)	
LCX	74 (19.0)	46 (16.5)	
Venous graft	17 (4.4)	1 (0.4)	
LM	1 (0.3)	0	
J‑CTO score			0.12
0 (easy)	39 (10.1)	23 (8.3)	
1 (intermediate)	118 (30.5)	88 (31.8)	
2 (difficult)	136 (35.1)	71 (25.6)	
≥ 3 (very difficult)	94 (24.3)	95 (34.3)	
*Procedural characteristics*			
Single access sites	275 (70.9)	137 (49.3)	< 0.01
Femoral	251 (91.3)	60 (47.3)	
Radial	24 (8.7)	74 (51.6)	
Dual access sites	113 (29.1)	144 (50.4)	< 0.01
Femoral/femoral	104 (92.0)	40 (27.8)	
Radial/femoral	7 (6.2)	100 (69.4)	
Hybrid approach used	39 (10.0)	68 (24.5)	< 0.001
Number of stents	2.3 ± 1.2	2.3 ± 1.0	0.88
Length of stent(s), mm	59.1 ± 33.8	66.4 ± 32.3	0.04
Procedure time, min	55.7 ± 34.4	70.3 ± 43.9	< 0.01
Fluoroscopy time, min	24.3 ± 19.7	26.6 ± 19.3	0.39
Wire crossing time, min	21.0 ± 17.6	26.0 ± 29.3	0.02
Radiation (DAP), Gy·cm^2^	134.2 ± 114.5	63.9 ± 57.0	< 0.001
Contrast amount, ml	225.3 ± 111.2	173.0 ± 78.8	< 0.001

Data are mean ± standard deviation or* n* (%)

^a^ Complete baseline, angiographic and procedural data are listed in Tables S2 and S3

*ACS* acute coronary syndrome, *CABG* coronary artery bypass grafting, *CTO* chronic total occlusion, *CVD* cardiovascular disease, *DAP* dose area product, *LAD* left anterior descending artery, *LCX* left circumflex artery, *LM* left main artery, *MDRD* Modification of Diet in Renal Disease, *MRI* magnetic resonance imaging, *PCI* percutaneous coronary intervention, *RCA* right coronary artery

### Outcomes

**Fig. 2:**
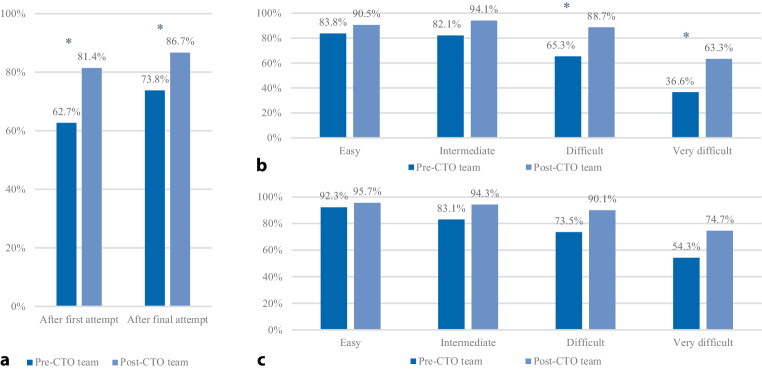
Angiographic success of percutaneous coronary intervention (*PCI*) for chronic total occlusion (*CTO*) in pre–CTO team and post–CTO team groups. **a** Success rates after first and final attempts. **b** Success rates after first attempt stratified by Multicentre CTO Registry of Japan (*J‑CTO*) score. **c** Success rates after final attempt stratified by J‑CTO score. Values are percentages of total number of procedures performed. ^a^indicates *p* ≤ 0.001

The angiographic success rate of the *first attempt *was significantly higher in the post–CTO team group than in the pre–CTO team group (81.4% vs 62.7%; *p* < 0.001). The angiographic success rate after the *final attempt* increased by 5.3% in the post–CTO team group and by 11.1% in the pre–CTO team group (86.7% vs 73.8%; *p* = 0.001). The success rate was significantly higher for CTO lesions classified as ‘difficult’ or ‘very difficult’ based on the J‑CTO score after the first and final attempt in the post–CTO team group than in the pre–CTO team group (Fig. 2).

**Fig. 3:**
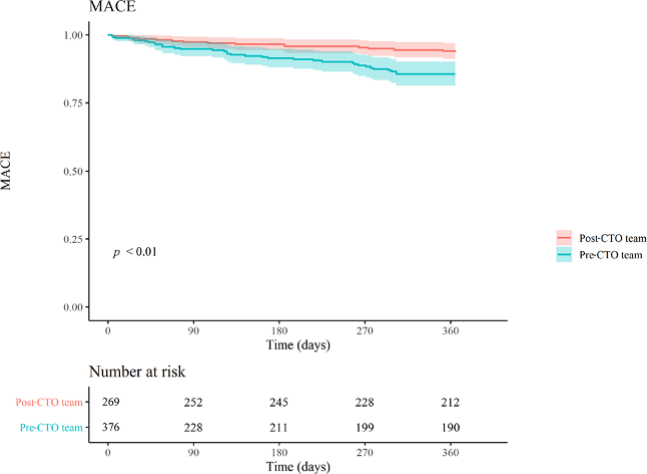
Major adverse cardiac event (*MACE*)–free survival after percutaneous coronary intervention (*PCI*) for chronic total occlusion in pre–CTO team and post–CTO team groups up to 1 year of follow-up

The total in-hospital complication rate was 7.3% with a higher major in-hospital complication rate in the post-CTO team group than in the pre-CTO team group (4.7% vs 1.7%; *p* = 0.02). However, the incidence of missing data on in-hospital MI was higher in the pre–CTO team group than in the post–CTO team group (45.6% vs 9.6%). Procedural or in-hospital MI occurred more often in the post–CTO team group than in the pre–CTO team group (3.2% vs 0.2%; *p* < 0.01) (Tab. 2). The incidence of MACE was significantly lower in the post–CTO team group during the first year after the intervention (6.4% vs 16.0%; *p* < 0.01) (Tab. 2 and Fig. 3). An additional Cox proportional hazard analysis was performed for potential confounders age, hypertension and assignment to post-CTO team group. The model showed that hypertension was not an independent predictor of MACE but age (hazard ratio (HR): 1.03; 95% confidence interval (CI): 1.01–1.06) and assignment to the post–CTO team group (HR: 0.37; 95% CI: 0.20–0.66). The angina pectoris classification (CCS) was significantly improved after 30 days of follow-up (*p* < 0.001) and was sustained after 1 year (*p* < 0.001) in both the pre–CTO team and post–CTO team groups compared with the pre-procedural CCS (see Fig. S1 in Electronic Supplementary Material).

**Table 2:** Outcomes

Variable	Pre–CTO team (389 procedures)	Post–CTO team (279 procedures)	*P*-value
Primary outcomes			
Angiographic success after first attempt	244 (62.7)	227 (81.4)	< 0.001
Angiographic success after final attempt	287 (73.8)	242 (86.7)	< 0.001
Secondary outcomes	*n* = 376	*n* = 269	–
*In-hospital and procedural complications/events*			
All-cause mortality	2 (0.4)	2 (0.6)	0.65
Urgent cardiac surgery	1 (0.2)	1 (0.3)	1.00
Urgent re-PCI	1 (0.2)	0	1.00
In-stent thrombosis	0	0	–
Coronary perforation	6 (1.3)	3 (0.9)	1.00
Dissection of target vessel	3 (0.6)	5 (1.6)	0.28
Dissection of donor vessel	0	3 (0.9)	0.06
Myocardial infarction	1 (0.2)	10 (3.2)	< 0.01
Pacemaker implantation	0	2 (0.6)	0.16
Stroke	0	0	–
Bleeding	13 (2.7)	6 (1.9)	0.46
Minor bleeding	5 (1.0)	2 (0.6)	
Major bleeding	8 (1.7)	4 (1.3)	
Cardiac tamponade	3 (0.6)	3 (0.9)	0.69
Cardiac arrhythmia^a^	1 (0.2)	3 (0.9)	0.31
*30-day follow-up*	*n* = 372	*n* = 269	–
All-cause mortality	4 (1.1)	0	0.14
Myocardial infarction	2 (0.6)	3 (1.1)	0.66
Target vessel revascularisation	3 (0.9)	0	0.26
MACE	6 (1.7)	3 (1.1)	0.74
*1‑year follow-up*	*n* = 370	*n* = 268	–
All-cause mortality	13 (3.5)	5 (1.9)	0.23
Myocardial infarction	10 (4.9)	9 (3.6)	0.50
Target vessel revascularisation	17 (5.1)	10 (3.8)	0.47
MACE	35 (16.0)	16 (6.4)	< 0.01

Data are *n* (%)

^a^ Includes ventricular arrhythmia in pre–CTO team group and atrioventricular block, sick sinus syndrome and asystole in post–CTO team group

*CTO* chronic total occlusion, *PCI* percutaneous coronary intervention, *MACE* major adverse cardiac event

In the post–CTO team group, a total of 115 serial baseline and 1‑year follow-up completed SF-36 forms were collected (response rate: 42.6%). In the group with angiographic successful CTO-PCI (*n* = 96), mean scores on all 8 QoL domains were significantly higher at 1‑year follow-up compared with the baseline scores (see Fig. S2 in Electronic Supplementary Material). In the group with angiographic unsuccessful CTO-PCI (*n* = 19), the mean score for physical functioning was significantly higher at 1‑year follow-up.

## Discussion

In this study, we demonstrated higher angiographic success rates and improved clinical outcomes of CTO-PCI after implementation of a dedicated CTO team. Centralising CTO-PCI procedures by assigning them to dedicated operators with expert training in the hybrid approach and complication management, using a systematic pre-procedural planning process, scheduling two interventional cardiologists (double scrub) for complex cases, and ensuring access to a state-of-the-art equipment set led to favourable clinical outcomes and an improvement in QoL in a more complex CTO population.

Our finding of improved angiographic success rates of 73.8% to 86.7% after the final attempt was in line with data from previous studies [5, 13, 14]. Syrseloudis et al. showed similar results in a single-centre study after introducing a novel technique (reversed controlled antegrade and retrograde subintimal tracking) that led to an increased procedural success rate in complex lesions [14]. In this study, the positive effect of the dedicated CTO team and hybrid approach was demonstrated by an increase in the success rate for each J‑CTO score, with significant differences for the difficult and very difficult CTOs. These results are similar to the outcomes of earlier studies in which the hybrid approach was used [4, 15, 16].

Interestingly, in our real-world study, we found a significant difference in the incidence of MACE at 1‑year follow-up after the establishment of the CTO team (16.0% in pre–CTO team group vs 6.4% in post–CTO team group). This difference could be partially explained by external factors such as the introduction of thin and ultrathin strut drug-eluting stents. However, we identified several factors related to the dedicated CTO team that were probably just as important: operator education and skills, introduction of new recanalisation techniques (antegrade dissection and re-entry using the CrossBoss/Stingray device), use of the hybrid approach, improvement in CTO materials and complication management.

Moreover, our results were highly favourable compared with recent data from a published registry study of all CTO-PCI procedures performed in the Netherlands. The authors reported an almost twice higher mortality rate (3.7% vs 1.9%) and a three times higher rate of TVR (11.3% vs 3.8%) after 1 year [1]. Other randomised CTO trials such as the EXPLORE and PRISON IV trials showed comparable MACE rates (5.4% after 4 months in EXPLORE trial and 7.0% after 1 year in PRISON IV trial) [8, 10]. In our study, symptom relief was demonstrated by a significant reduction in angina symptoms (CCS classification) at 30 days, which was sustained at 1‑year follow-up. These results were consistent with those of the EURO CTO trial, [9] which showed a significant increase in the number of asymptomatic patients in both groups, although this increase was significantly larger in the CTO-PCI group (PCI: 71.6% vs optimal medical therapy: 13.8%; *p* < 0.01) [9].

Our finding of a total in-hospital complication rate of 7.3% was also in line with earlier research. Several observational studies have shown comparable complication rates (4.3%–5.3%) [4, 13, 16]. In our study, the rate of major in-hospital complications was higher in the post–CTO team group than in the pre–CTO team group (4.7% vs 1.7%; *p* = 0.02) due to the increased incidence of periprocedural/in-hospital MI (3.2% vs 0.2%; *p* < 0.01). This could be explained by the following probable factors: transient ischaemia during a retrograde approach through collateral channel with a rise of biomarker levels, side-branch occlusions with the use of anterograde and retrograde dissection re-entry techniques in the post–CTO team group, and probable underestimation of the in-hospital complication rate with a higher incidence of missing data on in-hospital MI in the pre–CTO team group than in the post–CTO team group (45.6% vs 9.6%).

We observed baseline differences in mean age, hypertension, ischaemia confirmation and wall motion between the pre– and post–CTO team groups. The higher mean age of the post–CTO team group reflected a more complex CTO population. The differences in ischaemia confirmation and wall motion could be explained by a lower threshold for CTO-PCI in the presence of only viable myocardium on echocardiography [17].

Finally, some differences in angiographic characteristics between the pre– and post–CTO team groups could be clarified by the introduction of a new low-dose X‑ray system (AlluraClarity, Royal Philips, Amsterdam, the Netherlands) in November 2016. This new X‑ray system offered improved visualisation in combination with reduced radiation, which could explain the higher incidence of observed collaterals and mild calcifications and—on the other hand—a significant reduction in radiation exposure in the post–CTO team group. The standard use of dual access and bilateral contrast injection contributed to a shorter estimated occlusion length on angiography. Procedural time was increased by 5 min in the post–CTO team group because of higher complexity of CTO lesions and increased use of more than one (hybrid) technique per procedure. On the other hand, the amount of contrast used was significantly reduced by the increased use of the retrograde approach, which requires less contrast.

### Study limitations

This study was limited by its observational nature with the possibility of introducing potential confounders. Differences were observed in baseline characteristics such as age and hypertension between the groups. An additional analysis showed independent association of age and assignment to the post-CTO team to MACE. This analysis strengthened our overall findings on clinical outcomes by showing a lower MACE risk despite an incremental risk of events related to higher mean age in the post–CTO team group.

Furthermore, there were more missing data on clinical follow-up of MI (9.6% vs 45.6%) in the pre–CTO team group than in the post–CTO team group, which might have underestimated the event rate in the first group.

Another limitation of our study is the lower number of respondents included in the QoL analyses. As QoL questionnaires have been introduced for all PCI patients in 2018, they were only available for the post–CTO team group. Consequently, it was not possible to compare the pre–CTO team group with the post–CTO team group with regard to changes in QoL.

## Conclusion

In this real-world study, we showed significant improvement of angiographic success after the establishment of a dedicated CTO programme using the hybrid approach for CTO-PCI. This resulted in a significant improvement in clinical outcomes, angina symptom relief and QoL at 1‑year follow-up.

## Supplementary Information

Tab. S1 Angiographic and clinical endpoint definitions

Tab. S2 Complete overview of baseline and angiographic characteristics of pre–CTO team and post–CTO team groups

Tab. S3 Complete overview of angiographic and procedural characteristics of final procedures for pre–CTO team and post–CTO team groups (668 procedures)

Fig. S1 CCS classification progress after CTO-PCI

Fig. S2 Detailed description of angiographic definitions and characteristics, and treatment success
