# Supplementary material for: Improvement of angiographic and clinical outcomes of percutaneous coronary intervention for chronic total occlusion after implementation of a dedicated team: a single-centre experience
Source: Neth Heart J. 2022 Nov 29;31(3):117–23. doi: 10.1007/s12471-022-01732-5 (PMC9950300; doi:10.1007/s12471-022-01732-5)
Supplement: Supplementary file 1 — Tab. S1 Angiographic and clinical endpoint definitions [file 12471_2022_1732_MOESM1_ESM.docx]

**Tab. S1** Angiographic and clinical endpoint definitions

A CTO was defined as complete obstruction of the coronary arteries for at least three months (based on clinical and coronary angiography findings) with a Trombolysis in Myocardial Infarction (TIMI) grade 0 antegrade flow. Complexity of the CTO lesion was assessed by the J-CTO score [7]. Occlusion length was estimated from the index angiographic images in patients with bilateral approach or after recanalization and balloon inflation in procedures with single access approach. The degree of collateral size was classified according to the Werner classification of collateral size (CC classification). The degree of calcification was estimated on angiographic images without contrast injection in the coronaries. Angiographic success was defined as recovery of TIMI III grade antegrade flow in the target vessel and ≤ 30% residual stenosis of the target segment.

Angina complaints were defined following the Canadian Cardiovascular Society grading of angina pectoris (CCS) classification. Periprocedural complications included bleeding, MI, coronary perforation leading to cardiac tamponade or additional intervention, target or donor vessel dissection leading to additional intervention, cardiovascular death and cardiac arrhythmia. Minor bleeding was defined as a Bleeding Academic Research Consortium (BARC) score of 0-2 and major bleeding as a BARC score ≥ 3a including cardiac tamponade. Periprocedural/in-hospital MI were defined if at least two of the three following criteria were met: 1) prolonged chest pain ≥ 20 minutes, 2) enzyme changes (more than double the upper limits of Creatinine kinase (CK), CK-MB, or Relative Index, 3) ST-T-wave changes or new Q-waves on serial ECGs indicative of myocardial damage. Myocardial infarction during follow-up was defined as following the latest ESC guideline ‘Definition of Myocardial Infarction (2018) [11]. Target vessel revascularization (TVR) was defined as any unplanned PCI or surgical bypass of any segment of the target vessel. Major adverse cardiac events (MACE) was defined as composite of myocardial infarction, TVR and all-cause death in-hospital or during the follow-up period. Quality of life was assessed using the 36 items Short Form Health Survey (SF36 version 2) [12]. The questionnaire was completed at baseline (up to two months before the PCI) and 10 to 14 months after the PCI.
