# Supplementary material for: Improvement of angiographic and clinical outcomes of percutaneous coronary intervention for chronic total occlusion after implementation of a dedicated team: a single-centre experience
Source: Neth Heart J. 2022 Nov 29;31(3):117–23. doi: 10.1007/s12471-022-01732-5 (PMC9950300; doi:10.1007/s12471-022-01732-5)
Supplement: Supplementary file 2 — Tab. S2 Complete overview of baseline and angiographic characteristics of pre–CTO team and post–CTO team groups [file 12471_2022_1732_MOESM2_ESM.docx]

**Tab. S2** Complete overview of baseline and angiographic characteristics of pre–CTO team and post–CTO team groups

| Variables | Pre CTO-team  (n = 376) | Post CTO-team  (n = 269) | P value |
| --- | --- | --- | --- |
| Age, yrs | 64.2 ± 10.8 | 66.9 ± 10.3 | <0.01 |
| Male | 291 (77.6%) | 215 (79.3%) | 0.63 |
| Diabetes mellitus | 86 (22.9%) | 60 (22.1%) | 0.85 |
| Insulin-dependent | 29 (7.7%) | 25 (9.2%) | 0.51 |
| Current smoking | 81 (29.9%) | 55 (23.7%) | 0.13 |
| Hypertension | 244 (65.1%) | 220 (81.5%) | <0.01 |
| Hypercholesterolemia | 336 (89.6%) | 232 (86.2%) | 0.21 |
| Family history of CVD | 129 (55.6%) | 101 (60.1%) | 0.41 |
| Chronic kidney impairment* | 41 (11.0%) | 43 (15.9%) | 0.08 |
| Previous myocardial infarction | 179 (47.7%) | 111 (41.0%) | 0.09 |
| Previous PCI | 162 (43.2%) | 117 (43.2%) | 1.00 |
| Previous CABG | 87 (23.2%) | 62 (22.9%) | 1.00 |
| CABG target vessel | 59 (66.3%) | 38 (62.3%) | 0.73 |
| Previous peripheral artery disease | 28 (7.5%) | 26 (9.6%) | 0.39 |
| Previous stroke | 27 (7.2%) | 21 (7.7%) | 0.88 |
| Atrial fibrillation | 40 (10.7%) | 35 (12.9%) | 0.39 |
| Ischemia (confirmed) | 216 (57.6%) | 117 (43.2%) | <0.01 |
| Nuclear imaging | 171 (69.2%) | 88 (59.9%) | <0.01 |
| Bicycle test | 62 (25.1%) | 35 (23.8%) | 0.22 |
| MRI | 14 (5.7%) | 23 (15.6%) | 0.02 |
| Stress echo | 0 | 1 (0.7%) | 0.42 |
| Wall motion |  |  | < 0.01 |
| Normal | 152 (47.9%) | 160 (67.2%) | <0.01 |
| Hypokinetic | 84 (26.5%) | 55 (23.1%) | 0.38 |
| Akinetic | 81 (25.6%) | 23 (9.7%) | <0.01 |
| Wall motion test |  |  | <0.01 |
| Echo | 258 (81.6%) | 178 (74.5%) | 0.61 |
| Nuclear | 50 (15.8%) | 40 (16.7%) | 0.57 |
| MRI | 8 (2.5%) | 21 (8.8%) | <0.01 |
| Left ventricular function |  |  | 0.08 |
| > 50% | 272 (73.5%) | 209 (79.8%) | 0.07 |
| 35 – 50% | 65 (17.6%) | 41 (15.6%) | 0.59 |
| < 35% | 33 (8.9%) | 12 (4.6%) | 0.04 |
| Presentation |  |  | <0.01 |
| Stable angina | 313 (83.5%) | 213 (78.6%) | 0.13 |
| Unstable angina | 28 (7.5%) | 13 (4.8%) | 0.19 |
| ACS | 33 (8.8%) | 37 (13.7%) | 0.06 |
| Heart failure | 1 (0.3%) | 8 (3.0%) | <0.01 |

Values are *n* (%) or mean ± SD.ACS, acute coronary syndrome; CABG, coronary artery bypass grafting; CTO, chronic total occlusion; CVD, cardiovascular disease; MDRD, Modification of Diet in Renal Disease; MRI, magnetic resonance imaging; PCI, percutaneous coronary intervention.
