# Supplementary material for: Improvement of angiographic and clinical outcomes of percutaneous coronary intervention for chronic total occlusion after implementation of a dedicated team: a single-centre experience
Source: Neth Heart J. 2022 Nov 29;31(3):117–23. doi: 10.1007/s12471-022-01732-5 (PMC9950300; doi:10.1007/s12471-022-01732-5)
Supplement: Supplementary file 3 — Tab. S3 Complete overview of angiographic and procedural characteristics of final procedures for pre–CTO team and post–CTO team groups (668 procedures) [file 12471_2022_1732_MOESM3_ESM.docx]

**Tab. S3** Complete overview of angiographic and procedural characteristics of final procedures for pre–CTO team and post–CTO team groups (668 procedures)

| **Angiographic characteristic** | **Pre CTO-team**  **(389 procedures)** | **Post CTO-team**  **(279 procedures)** | **P value** |
| --- | --- | --- | --- |
| Occluded target vessel |  |  | <0.01 |
| RCA | 181 (46.5%) | 142 (50.9%) | 0.27 |
| LAD | 116 (29.8%) | 90 (32.3%) | 0.55 |
| LCX | 74 (19.0%) | 46 (16.5%) | 0.42 |
| Venous graft | 17 (4.4%) | 1 (0.4%) | <0.01 |
| LM | 1 (0.3%) | 0 | 1.00 |
| CTO lesion characteristics |  |  |  |
| Bridging collaterals | 132 (34.0%) | 113 (40.5%) | 0.09 |
| Retrograde collaterals | 281 (72.4%) | 239 (85.7%) | < 0.01 |
| Size collaterals |  |  | 0.18 |
| CC0 | 56 (14.4%) | 54 (19.4%) | 0.09 |
| CC1 | 234 (60.3%) | 151 (54.1%) | 0.13 |
| CC2 | 98 (25.3%) | 70 (25.1%) | 1.00 |
| In stent occlusion | 56 (14.4%) | 15 (5.4%) | <0.01 |
| Calcification |  |  | < 0.01 |
| Mild | 92 (23.7%) | 144 (51.6%) | <0.01 |
| Severe | 57 (14.7%) | 62 (22.2%) | 0.01 |
| Not evident | 239 (61.6%) | 71 (25.4%) | <0.01 |
| Bending >45 degrees | 124 (31.9%) | 86 (31.0%) | 0.87 |
| Occlusion length (mm) | 33.1 ± 22.0 | 23.0 ± 14.0 | < 0.01 |
| Retry | 88 (22.6%) | 54 (19.4%) | 0.34 |
| Severe/moderate tortuosity | 68 (17.5%) | 67 (24.3%) | 0.04 |
| J-CTO score |  |  | 0.12 |
| 0 = Easy | 39 (10.1%) | 23 (8.3%) | 0.50 |
| 1 = Intermediate | 118 (30.5%) | 88 (31.8%) | 0.80 |
| 2 = Difficult | 136 (35.1%) | 71 (25.6%) | 0.01 |
| ≥ 3 = Very difficult | 94 (24.3%) | 95 (34.3%) | <0.01 |
| Procedural characteristics |  |  |  |
| Arterial Access |  |  | < 0.001 |
| Single access sites | 275 (70.9%) | 137 (49.3%) | <0.01 |
| Femoral | 251 (91.3%) | 60 (47.3%) | <0.01 |
| Radial | 24 (8.7%) | 74 (51.6%) | <0.01 |
| Brachial | 0 | 1 (1.1%) | 0.33 |
| Dual access sites | 113 (29.1%) | 144 (50.4%) | <0.01 |
| Femoral / femoral | 104 (92.0%) | 40 (27.8%) | <0.01 |
| Radial / femoral | 7 (6.2%) | 100 (69.4%) | <0.01 |
| Radial / radial | 2 (1.8%) | 4 (2.8%) | 0.70 |
| Hybrid approach used | 39 (10.0%) | 68 (24.5%) | < 0.001 |
| Final technique |  |  | < 0.001 |
| AWE | 356 (91.8%) | 196 (70.3%) | <0.01 |
| ADR | 1 (0.3%) | 9 (3.2%) | <0.01 |
| RWE | 31 (8.0%) | 27 (9.7%) | 0.49 |
| RDR | 0 | 30 (10.8%) | <0.01 |
| Number of stents | 2.3 ± (1.2) | 2.3 ± (1.0) | 0.88 |
| Length of stent(s) (mm) | 59.1 ± 33.8 | 66.4 ± 32.3 | 0.04 |
| Procedure time (min) | 55.7 ± 34.4 | 70.3 ± 43.9 | <0.01 |
| Fluoroscopy time (min) | 24.3 ± 19.7 | 26.6 ± 19.3 | 0.39 |
| Wire crossing time (min) | 21.0 ± 17.6 | 26.0 ± 29.3 | 0.02 |
| Radiation (DAP) (Gy·cm^2^) | 134.2 ± 114.5 | 63.9 ± 57.0 | < 0.001 |
| Contrast (ml) | 225.3 ± 111.2 | 173.0 ± 78.8 | < 0.001 |
| Angiographic success after first attempt | 244 (62.7%) | 227 (81.4%) | < 0.001 |
| Angiographic success after final attempt | 287 (73.8%) | 242 (86.7%) | < 0.001 |

Values are *n* (%) or mean ± SD. CC classification, Werner classification of collateral size; CTO, chronic total occlusion; DAP, dose area product in mGym2; Gy·cm^2^, gray-centimetres squared; J-CTO score, Multicentre Chronic Total Occlusion Registry of Japan scoring; LAD, left anterior descending artery; LCX, left circumflex artery; LM, left main artery; min, minutes; ml, millimetre; mm, millimetre; RCA, right coronary artery;
