# Supplementary material for: Improvement of angiographic and clinical outcomes of percutaneous coronary intervention for chronic total occlusion after implementation of a dedicated team: a single-centre experience
Source: Neth Heart J. 2022 Nov 29;31(3):117–23. doi: 10.1007/s12471-022-01732-5 (PMC9950300; doi:10.1007/s12471-022-01732-5)
Supplement: Supplementary file 4 — Fig. S1 CCS classification progress after CTO-PCI [file 12471_2022_1732_MOESM4_ESM.docx]

**Fig. S1** CCS classification progress after CTO-PCI

*CCS, Canadian Cardiovascular Society grading of angina pectoris*

*P value: pre-CTO team group pre-procedural vs. 30-days p< 0.001; pre-procedural vs. one-year p< 0.001. Post-CTO team group start vs. 30-days p< 0.001; start vs. one-year p< 0.001*
