# Supplementary material for: Improvement of angiographic and clinical outcomes of percutaneous coronary intervention for chronic total occlusion after implementation of a dedicated team: a single-centre experience
Source: Neth Heart J. 2022 Nov 29;31(3):117–23. doi: 10.1007/s12471-022-01732-5 (PMC9950300; doi:10.1007/s12471-022-01732-5)
Supplement: Supplementary file 5 — Fig. S2 Detailed description of angiographic definitions and characteristics, and treatment success [file 12471_2022_1732_MOESM5_ESM.docx]

**Fig. S2** Effect of treatment success on quality of life

*p < 0.01*

*Successful CTO-PCI*

*p < 0.01*

*QoL, quality of life; SF-36, Short Form Health survey (36 items)*

*5A. Change in quality of life after angiographic successful CTO-PCI; 5B. Change in quality of life after angiographic unsuccessful CTO-PCI*

*P-value: 5A: p< 0.01 for all domains. 5B: p< 0.01 for physical functioning. p> 0.05 for the other domains*

5B.

*Unsuccessful CTO-PCI*

*p > 0.05*

5A.
